# Dietary Sodium and Nonalcoholic Fatty Liver Disease: A Systematic Review

**DOI:** 10.3390/antiox12030599

**Published:** 2023-02-28

**Authors:** Guilherme da Silva Ferreira, Sergio Catanozi, Marisa Passarelli

**Affiliations:** 1 Laboratorio de Lipides (LIM-10), Hospital das Clinicas (HCFMUSP) da Faculdade de Medicina da Universidade de São Paulo, Sao Paulo 01246-000, Brazil; 2 Programa de Pós Graduação em Medicina, Universidade Nove de Julho, Sao Paulo 01525-000, Brazil

**Keywords:** sodium consumption, dietary sodium, nonalcoholic fatty liver disease, steatosis

## Abstract

(1) Introduction: Restriction in sodium intake is an important strategy for reducing cardiovascular morbidity and mortality, considering the direct influence of high-sodium diet consumption on the development of hypertension and cardiovascular diseases. There are only a few studies dealing with the influence of dietary sodium on the development of nonalcoholic fatty liver disease (NAFLD). In this systematic review, evidence in humans and animal models was compiled in a critical view of the influence of dietary sodium intake patterns on NAFLD markers; (2) Methods: Systematic review of PubMed data. Clinical outcomes included the prevalence/incidence of NAFLD for human studies, and NAFLD markers (hepatic lipogenesis, and markers of steatosis, fibrosis, and inflammation) for animal studies. The protocol was registered at the International Prospective Register of Systematic Review (PROSPERO; CRD42023390447); (3) Results and Conclusion: Seven studies in humans and eight in animals were included. All studies in humans were observational and associated high-sodium intake with NAFLD. However, in animals, both the increased and reduced consumption of sodium negatively influenced markers of liver steatosis, inflammation, and fibrosis.

## 1. Introduction

Non-alcoholic fatty liver disease (NAFLD) is represented by a continuum that begins with steatosis, progressing in a small percentage of subjects to steatohepatitis, cirrhosis, and cellular hepatocarcinoma. It is the liver disease with the highest global incidence and a prevalence that comprises around 25% of the world’s population [1,2]. The pathogenesis of NAFLD is complex, and many factors contribute to its development. Obesity is one of the most important risk factors. In addition, other metabolic diseases, environmental conditions, and genetics add to its genesis and evolution. Insulin resistance, inflammation, lipotoxicity, endoplasmic reticulum stress, and mitochondrial dysfunction are the basis of the pathophysiology of NAFLD, which can be well managed by healthy dietary habits.

Elevated sodium intake is related to the development of many metabolic disturbances. In particular, it increases blood pressure and the prevalence of stroke and myocardium infarction. On the other hand, dietary sodium restriction is a nonpharmacological strategy that allows for the reduction of blood pressure and its related comorbidities [3]. Nonetheless, a more severe restriction in sodium intake has been reported as having adverse negative effects on lipid and glucose homeostasis by the activation of the renin–angiotensin–aldosterone system (RAAS), increased insulin resistance, inflammation, and triglyceride (TG) accumulation in the liver [4].

There are few human and animal studies dealing with the influence of sodium consumption on the development of NAFLD. Shojaei-Zarghani et al. [5], in a systematic review and meta-analysis of observational studies, found that people with a high-sodium intake have a 60% greater risk of developing NAFLD compared to those with low consumption. Xu and Mao [6] reviewed the potential mechanisms for this relationship: elevation of caloric consumption, hyperosmolarity induced by salt, insulin resistance, endogenous fructose synthesis, and dysfunction of the RAAS.

Conversely, animal studies have also observed an increase in NAFLD markers, such as hepatic TG concentration and inflammatory cytokines [7,8]. None of the systematic reviews included animal studies in their analyses. Thus, the objective of this review is to update/compile and critically analyze, in a narrative way, research on humans and animals that verified the influence of different patterns of sodium consumption on NAFLD markers.

## 2. Materials and Methods

The Preferred Reporting Items for Systematic reviews and Meta-Analyses (PRISMA) statement, published in 2009 (PRISMA; http://www.prisma-statement.org/; accessed on 26 January 2022), was utilized, and the protocol was registered at the International Prospective Register of Systematic Review (PROSPERO; CRD42023390447).

### 2.1. Data Research

Data research was performed by using the PubMed database on 17 July 2021, with the following search strategy: ((Sodium Chloride) OR (Sodium Chloride, (22)Na) OR (Sodium Chloride, (24)NaCl) OR (Diet, Sodium-Restricted) OR (Diet, Sodium Restricted) OR (Diets, Sodium-Restricted) OR (Sodium-Restricted Diet) OR (Sodium-Restricted Diets) OR (Diet, Low-Sodium) OR (Diet, Low Sodium) OR (Diets, Low-Sodium) OR (Low-Sodium Diet) OR (Low-Sodium Diets) OR (Diet, Low-Salt) OR (Diet, Low Salt) OR (Diets, Low-Salt) OR (Low-Salt Diet) OR (Low-Salt Diets) OR (Diet, Salt-Free) OR (Diet, Salt Free) OR (Diets, Salt-Free) OR (Salt-Free Diet) OR (Salt-Free Diets) OR (Dietary Sodium) OR (Sodium Chloride, Dietary) OR (Table Salt) OR (Salt, Table) OR (Dietary Sodium Chloride) OR (Chloride, Dietary Sodium)) AND ((Non-alcoholic Fatty Liver Disease) OR (Non alcoholic Fatty Liver Disease) OR (NAFLD) OR (Nonalcoholic Fatty Liver Disease) OR (Fatty Liver, Nonalcoholic) OR (Fatty Livers, Nonalcoholic) OR (Liver, Nonalcoholic Fatty) OR (Livers, Nonalcoholic Fatty) OR (Nonalcoholic Fatty Liver) OR (Nonalcoholic Fatty Liv-ers) OR (Nonalcoholic Steatohepatitis) OR (Nonalcoholic Steatohepatitides) OR (Steatohepatitides, Nonalcoholic) OR (Steatohepatitis, Nonalcoholic)).

Original studies that sought to understand the influence of sodium consumption on NAFLD, published in peer-reviewed scientific journals, were selected for the systematic review. They could be in humans (without the restriction of the population studied) or animals, with any experimental design, without the restriction of the publication date. They should compare the prevalence/incidence of NAFLD, or, if intervention, the alteration of steatosis or fibrosis markers in the liver according to sodium intake. In humans, sodium consumption should have been assessed by a food frequency questionnaire, 24-h recall, 24-h urine collection, or urine collection <24 h. NAFLD should have been assessed by biopsy, FibroScan, ultrasound, or validated formulas. Those studies that reported only the dietary pattern of people with NAFLD without correction for confounding factors, even though sodium intake was compared between cases and the control, were not selected. Studies that examined the impact of sodium intake on the same population as in previous research were also excluded. For any questions regarding inclusion and exclusion, the authors reached a consensus.

### 2.2. Screening, Extraction, and Synthesis of Data

**Figure 1:**
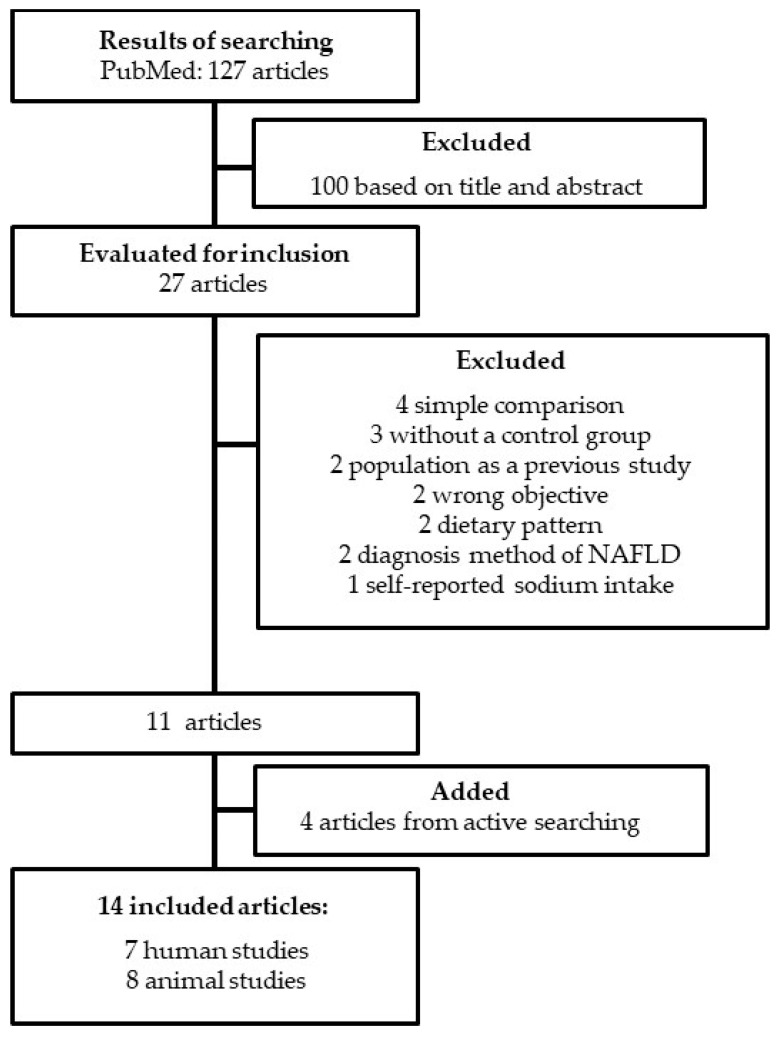
Research flow diagram of the screening and selection of studies.

For the screening of references and data extraction, the websites https://www.rayyan.ai/ [9] and https://srdrplus.ahrq.gov/ (accessed on 17 June 2021) were used. A total of 127 articles were identified in the literature search. They were initially screened based on the title and abstract. A more detailed analysis was performed on 27 articles. Of these, 11 were selected for inclusion in the systematic review. Additionally, 4 articles were added that did not appear in the systematic search, but were related to the objective of the review (one article was not indexed in Pubmed [10]; three articles were not found in the systematic search [8,11,12]). The flowchart of the screening and selection of the articles is demonstrated in Figure 1.

The data extracted from the articles were: author, year, study design, population studied, country, year of assessments, number of people, age, gender distribution, body mass index, method of assessment of sodium consumption and NAFLD, correction of sodium consumption by energy consumption, and results. In animal studies, data were extracted, including animal model, lineage, sex, age, dietary sodium composition, other dietary interventions, duration of the protocol, and results referring to metabolic variables (weight, blood glucose, plasma lipids), hepatic lipogenesis, and markers of steatosis, fibrosis, and inflammation.

Bias risk was assessed by the questionnaire “Joanna Briggs Institute (JBI) Critical Assessment Tool” in human studies (https://reviewersmanual.joannabriggs.org, accessed on 21 June 2021). Table 1 shows the questions and criteria used to judge biases using the JBI. In animal articles, the risk of bias was not evaluated using a standardized tool.

Data are summarized descriptively, guided by the extracted data described above and the judgment of biases (human studies).

## 3. Results

### 3.1. Animal Studies

The characteristics and results of animal studies are summarized in Table 2, Table 3 and Table 4. Eight articles were included in the review and descriptive analysis, with nine different studies (considering that Cabrera et al. performed experiments in two different models [7]). The results presented refer to comparisons between models with variations in sodium consumption, with other components of the diet being similar. If the authors have performed other interventions, these will not be discussed here.

Intervention protocols varied considerably between studies, making comparisons difficult. The main experimental model used was wild-type C57BL/6 (6/9) mice. Other models used were LDLr KO mice, ApoE KO/LOX-1 KO mice, and rats, each once. Interventions in sodium consumption lasted between 6 and 30 wk (Table 2), and, in general, were associated with other dietary modifications, such as a high-fat diet, deficiency in methionine-choline, together with sucralose or starch (Table 3). Steatosis, fibrosis, and inflammation were evaluated using biochemical and histological methods, mRNA quantification, and the levels of the disease marker proteins (Table 4).

#### 3.1.1. Effect of Increased Sodium Intake on Markers of Lipid Accumulation, Inflammation and Fibrosis in the Liver

Uetake et al. [13], in LOX-1 overexpressing hypercholesterolemic mice fed a high-fat diet containing 3.2% Na^+^ or chow diet containing 0.2% Na^+^ for 8 wk, observed an increase in score activity of NAFLD, liver fibrosis (plasma hyaluronic acid, liver fibronectin, and histological analysis), and inflammation (TNF) by the high-sodium diet. Liver TG were similar between the groups. The author attributed the deleterious effects of high-sodium intake to the increased oxidative stress since the administration of the antioxidant Tempol prevented NAFLD development.

Lanaspa et al. [14] added 1% NaCl to water containing 0.04% sucralose for 30 wk and observed that salt enrichment increased mice food consumption, weight gain, epididymal adipose tissue, insulin resistance, ALT, and AST. In the liver, sodium consumption increases TG deposition. Similar results were observed in mice that were given water containing 15% fructose and 1% NaCl, compared to those given only fructose. The authors associated these results with greater activation of the aldose reductase pathway, favoring endogenous fructose synthesis. Blocking fructose metabolism by knocking out fructokinase reversed most of the results described above, especially by decreasing hepatic TG. In resume, increased salt intake leads to elevated serum osmolality, activating the polyol pathway and resulting in the production of fructose. When fructose is metabolized by fructokinase, it triggers oxidative stress, leading to fat accumulation and decreased insulin sensitivity in the liver and leptin resistance in the hypothalamus. Leptin resistance contributes to liver steatosis by increasing appetite, and, possibly, decreasing energy expenditure.

**Table 2:** Summary of the characteristics of animal studies.

Authors (Year)	Animal Model	Age (wks)	% of Sodium in Diet	Other Changes in Diet	Time of Intervention (wks)
Xavier et al. (2003) [11]	Rats	After weaning	LS = 0.06% Na^+^ NS = 0.5% Na^+^	-	12
Uetake et al. (2015) [13]	ApoE KO/LOX-1 KO mice	8	NS = 0.2% Na^+^; HS = 3.2% Na^+^	High fat	8
Kim et al. (2017) [15]	C57BL/6J mice	32	NS1 = 0.14% Na^+^; NS2 = 0.4% Na+	High fat	13
Lanaspa et al. (2018) [14]	C57BL/6J mice	8	HS = 0.4% Na^+^ in drinking water	0.04% sucralose in drinking water	30
Do et al. (2020) [16]	C57BL/6J mice	6	NS = 0.2% Na^+^; HS = 1.6% Na^+^	Gelatinized starch	8
Cabrera et al. (2021) [7]	C57BL/6J mice	10	LS = 0.03% Na^+^; NS = 0.3% Na^+^; HS = 3% Na^+^	High-fat diet	12
Cabrera et al. (2021) [7]	C57BL/6J mice	10	LS = 0.03% Na^+^; NS = 0.3% Na^+^; HS = 3% Na^+^	Choline/methionine deficient diet	6
Ferreira et al. (2021) [8]	LDLr KO mice	12	LS = 0.06% Na^+^; NS = 0.5% Na^+^	-	12
Gao et al. (2022) [17]	C57BL/6 mice	6	NS = 0.4 Na+; HS = 8% Na^+^	-	16

ApoE KO = apolipoprotein E knockout; LDLr KO = LDL receptor knockout; LOX-1 KO = Lectin-like oxidized low-density receptor-1 knockout; Kim et al. (2017) did not comment on the amount of NaCl in the chow diet (based on the supplier company’s website it contains 0.34% NaCl/weight); in the study by Kim et al. (2017), 8-week-old mice started the study with a 6-month high-fat diet.

**Table 3:** Summary of the impact of sodium consumption on metabolic variables in animal studies.

Authors (Year)	Food Consumption	Body Mass or Weight Gain	Fasting Glycemia	Insulin Resistance	Plasma Lipids
High vs. normal sodium intake					
Uetake et al. (2015) [13]	-	-	-	-	-
Lanaspa et al. (2018) [14]	↑	↑	-	↑	
Do et al. (2020) [16]	=	=	-	-	= CT, TG, and LDLc
Gao et al. (2022) [17]	=	=	-	-	-
Low vs. normal sodium intake					
Xavier et al. (2003) [11]	=	LS = ↑ in the 2nd month (not in the 3rd)	=	-	
Kim et al. (2017) [15]	=	=	=	-	
Ferreira et al. (2021) [8]	=	↑	↑	↑	↑ TG
Normal and high vs. normal sodium intake					
Cabrera et al. (2021) [7]	=	HS ↓ *^†^	HS = ↓ *	HS = ↓ *^†^	-
Cabrera et al. (2021) [7]	-	=	-	=	-

* compared with normal-sodium; † compared with low-sodium; TC = total cholesterol; TG = triglycerides; LDLc = Low-density lipoprotein cholesterol; HS = high-sodium; LS = low-sodium.

**Table 4:** Summary of the effects of sodium consumption on markers of NAFLD.

Authors (Year)	TG Levels and Synthesis	Inflammation	Fibrosis	NAFLD Score	Mechanisms
High vs. normal sodium intake					
Uetake et al. (2015) [13]	= TG level	↑ TNF	↑	↑	Oxidative stress
Lanaspa et al. (2018) [14]	↑ TG level (histology and biochemical)	-	-	-	Activation of the aldose reductase pathway
Do et al. (2020) [16]	= TG level; = SREBP, ACC and FAS	↑ TNF, MCP-1, IL6	-	-	-
Gao et al. (2022) [17]	↑ TG level (histology and biochemical)	↑ (histology and mRNA of several citokines)	↑ (histology and mRNA of several proteins)	↑	Reduction of SIRT3
Low vs. normal sodium intake					
Kim et al. (2017) [15]	= steatosis (histology)	↓ *Il1b, Cxcl2* mRNA	↓ *Col1a1* mRNA	=	-
Xavier et al. (2003) [11]	↑ lipogenesis; = TG content	-	-	-	Increase in the uptake of fatty acids
Ferreira et al. (2021) [8]	↑ TG level (biochemical)	Not different: *Il6* and *Il10*	-	-	Metabolic impairment
Normal and high vs. normal sodium intake					
Cabrera et al. (2021) [7]	HS = ↓ TG level *^†^ (histology and concentration); HS = ↓ ACC^†^, FAS^†^, SCD1 *^†^ mRNA;	LS = ↑ TNF ^$^ and MCP1 *^$^ mRNA	LS = ↑ TIMP1 mRNA *^$^; HS = ↑ MMP9 and MMP13 mRNA *^†^	-	HS -↓ Aldosterone and mineralocorticoid receptor activation
Cabrera et al. (2021) [7]	HS ↓ FAS *^†^ mRNA; HS = ↓ TG level (histology and biochemical) *^†^	= MCP1	= TIMP1	-	-

* compared with normal-sodium; † compared with low-sodium; $ compared with high-sodium; TG = triglycerides; HS = high-sodium; LS = low-sodium; ACC = acetyl-CoA carboxylase; FAS = fatty acid synthase; IL6 = interleukin 6; IL10 = interleukin 10; MMP9 = matrix metalloproteinase 9; MMP13 = matrix metalloproteinase 13; TIMP1 = metallopeptidase Inhibitor 1; SIRT3 = sirtuin 3. Cabrera et al. (2021) result of the fibrosis content differs between the figure and the text; Kim et al. (2017) evaluated two concentrations of sodium in the diet considered normal; the one with the lowest concentration of sodium being ascribed as low.

Do et al. [16], fed wild-type C57BL/6J mice with gelatinized wheat starch with normal-sodium (0.2 Na^+^) or supplemented with 1.6 Na^+^ for 8 wk. Gelatinized wheat starch, per se, reduced plasma lipids, white adipose tissue weight, and hepatic fat deposition compared to the group that received a diet rich in wheat starch. When comparing the groups receiving gelatinized wheat starch with different concentrations of salt, there was an increase in the concentration of inflammatory proteins (TNF, MCP1, and IL6) in the liver but no differences in body weight gain, food consumption, plasma lipids (TC, TG, LDLc), and concentration of fat deposition in the liver.

Recently, Gao et al. [17] evaluated the effects of a 16 wk high-sodium diet (8% Na^+^) on the development of NAFLD in C57BL/6 mice. A group of animals switched from a high to a normal-sodium diet (0.4% Na^+^) at the middle point of the intervention. Although sodium overload did not interfere with body weight gain or food consumption, it stimulated systemic inflammation, liver damage (higher ALT and AST), and the development of NAFLD (higher steatosis, fibrosis, and NAFLD score). Furthermore, the livers of animals fed a high-sodium diet showed mitochondrial dysfunction, higher concentrations of TG, total cholesterol, and free fatty acids, and greater infiltration of inflammatory cells. Interestingly, all these effects persisted for at least 8 wk after the removal of diet, indicating a metabolic memory. Using a series of genetic manipulation strategies, the authors found that the effects of sodium, especially those related to memory, were attributed to epigenetic modulation, including increased acetylation of H3K27, which reduces sirtuin 3 (SIRT3) and increases inflammatory cytokine expression.

Contrary to the findings described above, Cabrera et al. [7] observed in two different animal models a positive effect of a high-sodium intake on NAFLD markers. In wild-type C57BL/6J mice fed a high-fat diet, high-sodium intake (3% Na^+^) for 12 wk reduced body weight gain, insulin resistance, and plasma glucose compared to the group fed a normal-sodium (0.3% Na^+^) or low-sodium (0.03% Na^+^) diet. Moreover, in the liver, the group with the highest sodium intake had a lower hepatic TG level (measured by histology or concentration/g of liver), expression of SCD1, a protein associated with TG synthesis, and an increase in matrix metalloproteinases (MMP9 and MMP13), proteins negatively associated with the presence of fibrosis, in comparison to animals that received a normal-sodium diet. The expression of the lipogenic enzymes ACC and FAS, and of TNF was reduced by the high-sodium diet as compared with the low-sodium group. In animals fed a methionine-choline deficient diet, a model of NAFLD induced by inflammation and fibrosis, the results were similar. The authors proposed that the findings were related to the diminished activation of the mineralocorticoid receptor due to the lower availability of plasma aldosterone, which allows for the higher expression of salt-inducible kinase1 (SIK1) responsible for downregulating lipogenesis. Previous studies have shown that mineralocorticoid receptor blockade is associated with the attenuation of steatosis and fibrosis induced by a methionine-choline-deficient diet [18].

#### 3.1.2. Effect of Sodium Intake Restriction on Markers of Inflammation and Fibrosis, and Lipid Accumulation in the Liver

In wild-type, C57BL/6J mice fed a high-fat diet for 6 months followed by a high-fat diet added with salt (1% NaCl; 0.4% Na^+^) or not (0.3% NaCl; 0.13% Na^+^) (both diet have an amount of sodium generally classified as normal), here Kim et al. [15] found at the end of the protocol similar body weight, fasting glucose, and food intake between groups, but increased plasma insulin, and ALT levels in the group consuming 0.4% Na^+^ than 0.13% Na^+^ diet. Sodium intake did not influence the assessment of steatosis, inflammation, ballooning, and NAFLD score. Nonetheless, the expression of inflammatory cytokines (*IL1b* and *Cxcl2*) and marker of stellate cell activation-collagen 1 (*Col1a1*)-were lower in the group with less sodium intake. The small difference between the content of sodium could limit the effect of this micronutrient; however, it may better simulate the difference in sodium consumption in humans’ diets.

Xavier et al. [11] studied rats on a normal-sodium (0.5% Na^+^) or low-sodium (0.06% Na^+^) diet for 12 wk and observed greater body weight gain in the 2nd month (but not in the 3rd month), increased retroperitoneal adipose tissue, and hepatic lipogenesis in mice with reduced sodium intake. Despite the increase in hepatic lipid synthesis, liver TG content was not different between the groups. These results were attributed to an increase in the uptake of fatty acids from the circulation and a reduction in energy expenditure in the low-sodium group.

The study by Cabrera et al. [7] cited above, despite mainly showing a protective effect of high-sodium intake on NAFLD, presents some data in the group fed a low-sodium diet. Low-sodium intake did not induce differences in relation to the normal sodium group in terms of body weight gain, fasting glycemia, insulinemia, insulin resistance, lipid synthesis, and hepatic TG concentration. On the other hand, it increased the expression of inflammatory cytokine (MCP-1), TIMP-1 (an inhibitor of matrix metalloproteinases), and diacylglycerol and esterified cholesterol in the liver.

Finally, Ferreira et al. [8] observed in hypercholesterolemic LDL receptor knockout mice (LDLr KO) that a low-sodium diet (0.06% Na^+^) for 12 wk, as compared to a normal-sodium diet, increased body weight, insulin resistance, and plasma TG levels. In the liver, the low-sodium diet induced the accumulation of TG, with no changes in the expression of *Acaca Fasn*, *Srebf1*, *Il6*, and *Il10*. Moreover, chronic sodium restriction increased the expression of markers of liver insulin resistance (*G6pc* and *Pck1*) and reduced the expression of transcription factors associated with oxidative metabolism (Prkaa2 and *Ppara*). Nonetheless, histological or biochemical markers of NAFLD were not addressed in that study.

### 3.2. Human Studies

The characteristics and results of the human studies are summarized in Table 5. Seven studies, carried out in six different countries (USA, Netherlands, Iran, South Korea, and Japan) since 2015, were included in the review and descriptive analysis. All of them had an observational and cross-sectional research design, and from 310 to 100,177 participants were included.

Zhou et al. [19] and Huh et al. [20] used data from national studies with random sampling of the non-institutionalized adult population (NHANES and KNHANES). Choi et al. [21], Emamat et al. [10], Takahashi et al. [22], and Luo et al. [12] collected a convenience sample from people who went to hospitals/clinics to carry out health assessments. The study by Emamat et al. [10] was designed to be case-control with patients with pancreaticobiliary disorders [23]. Takahashi et al. [22] conducted the study only in patients with type 2 diabetes. Van den Berg et al. [24] used a subpopulation of the Prevention of Renal and Vascular End-Stage Disease (PREVEND) study, a case-control research study that was selected from among the inhabitants of Groningen, Netherlands, random people (28 to 75 years old) with urinary albumin <10 mg vs. urinary albumin, the latter group having twice as many participants.

Only one study assessed sodium intake by the gold standard method, 24-h urinary sodium excretion. Urinary sodium excretion <24 h, food frequency questionnaire (FFQ), and 24-h dietary recall were used in three, two, and one studies, respectively (Table 5). Zhou et al. [19] and van den Berg [24] used data from more than one consumption assessment. Studies that evaluated sodium consumption by questionnaire corrected their values for energy consumption, either by the density or residual method.

The diagnosis of NAFLD was defined based on predictive formulas in four studies. The formulas used were the HSI (hepatic steatosis index), FLI (fatty liver index), and the NAFLD fibrous score (Table 5). Ultrasonography and FibroScan were performed twice and once, respectively (Table 5). No studies have evaluated 24-h urinary sodium excretion and ultrasound or elastography in the same subjects.

Data on sodium consumption, age, BMI, and percentage of women in the groups with the lowest and highest sodium intakes are described in Table 6. Luo et al. [12] did not present these data according to sodium consumption in the article. Each author decided how to perform the separation of people by sodium intake, which was either by median, tercile, quartile, or quintile. Table 6 demonstrates that age does not seem, systematically, to differ between groups with higher and lower sodium consumption. On the other hand, the group with the highest sodium intake had a higher frequency of elevated BMI and a lower percentage of the female sex compared to the group with the lowest sodium intake (Table 6).

The association between the prevalence of NAFLD and sodium intake was evaluated using multiple logistic or Poisson regression. All studies showed a positive association between sodium intake and the presence of NAFLD (Table 5). In some articles, the addition of BMI or percentage of body fat reduced or removed the statistical significance between sodium consumption and NAFLD [19,21]. Emamat et al. [10] when stratifying by BMI< or >25 kg/m^2^, observed no effect of sodium consumption on the prevalence of NAFLD in people with BMI < 25 kg/m^2^.

#### Bias Risk in Human Studies

The Table 7 presents, for the human studies, the judgments for the questions asked in the “Joanna Briggs Institute (JBI) critical evaluation tool”, a questionnaire to assess the risk of bias. On this topic, attention is drawn to the design of the studies. Cross-sectional studies themselves constitute an important risk of bias and are not suitable for assessing cause and effect.

The methods for diagnosing NAFLD and evaluating sodium consumption were mostly estimated using indirect methods, which can add great bias to the conclusions of the studies. Only a study by van den Berg et al. [24] performed a gold standard method to assess sodium intake. In addition, sodium intake varies over the days, requiring multiple assessments to reduce intra-individual error in the analysis, even when assessed using 24-h urinary sodium excretion. The diagnosis of NAFLD was performed using doubly indirect methods (HSI and FLI) in five of the eight surveys, while three used more accurate methods for NAFLD detection, such as ultrasound and FibroScan.

The control of confounders is essential, especially concerning energy intake and BMI. Moreover, other nutritional components, physical exercise, cardiometabolic diseases, and liver diseases must be controlled, given the relationship of these variables with both sodium intake and NAFLD. Zhou et al. [19] and Choi et al. [21] added the metabolic variables (BMI or obesity, blood pressure or hypertension, HOMA-IR, diabetes, and dyslipidemia) separately to the complete model instead of treating these factors together. van den Berg et al. [24], Luo et al. [12], and Takahashi et al. [22] did not include some of the following covariates in the statistical model: BMI, energy intake, dietary data, physical activity, liver disease, and sociodemographic data. The authors did not detail the method for choosing the variables included in the statistical models.

**Table 5:** Summary of characteristics and results from human studies.

Authors (Year of Publication)	Zhou et al. (2021) [19]	van den Berg et al. (2019) [24]	Choi et al. (2016) [21]	Huh et al. (2015) [20]	Authors (Year)	Emamat et al. (2021) [10]	Takahashi et al. (2022) [22]	Luo et al. (2022) [12]
Design	Cross-sectional	Cross-sectional	Cross-sectional	Cross-sectional	Design	Cross-sectional	Cross-sectional	Cross-sectional
Country of origin	USA	Netherlands	South Korea	South Korea	Country	Iran	Japan	China
Population	Non-institutionalized adults (>20 yo)	Adults with macro albuminuria and controls	Healthy adults	Non-institutionalized adults (>25 yo)	Population	People with NAFLD and controls with pancreaticobiliary disorders	Type 2 Diabetes	Adults (18–59 yo)
Year of collection	2007–2017	2001-2003	2011–2013	2010–2013	Year of collection	2015	2016–2018	2017–2019
n (total)	11,022	6132	M = 46.596; F = 53.581	27,433	n (total)	999	310	23,867
Sodium intake (method)	24-h food recall (2 evaluations)	24-h uNa^+^ (2 evaluations)	FFQ	Tanaka’s formula	Sodium intake (method)	FFQ	Tanaka’s formula	Tanaka’s formula
NAFLD diagnosis	Predictive formulas	Predictive formulas	Ultrasound	Predictive formulas	NAFLD diagnosis	FibroScan	Predictive formulas	Ultrasound
Cutoff points	HSI > 36	HSI > 36 and FLI > 60	-	HSI >= 35 and FLI > 60	Cutoff points	CAP > 263 and fibrosis score > 7 (db/m)	HSI ≥ 36	-
Results Multiple regression (OR or PR [95% CI])	with BMI (without HAS)–HIS: Q4 vs. Q1 = 1.30 (1.04; 1.64)	For each SD of sodium (55.99 mmol/L)–HSI = 1.40 (1.31–1.51); FLI = 1.30 (1.21; 1.41)	Q5 vs. Q1 (with BMI): Male: 1.16 (1.10, 1.22); Female: 1.11 (0.99, 1.24)	T3 vs. T1-HSI = 1.39 (1.26–1.55); FLI: = 1.29 (1.39–2.20). For each SD-HSI = 1.21 (1.16; 1.26); FLI = 1.29 (1.19; 1.41)	Results Multiple regression; (OR or PR [95% CI])	T3 vs. T1, = 2.42 (1.13–5.15)	>9.5 g/day vs. <9.5 g/day sodium = 1.76 (1.02–3.03)	Q4 vs. Q1 = 1.60 (1.47–1.76)

BMI = body mass index; HSI = hepatic steatosis index; FLI = fatty liver index; M = male; F = female; CAP = controlled attenuation parameter; OR = odds ratio; PR = prevalence ratio; CI = confidence interval; SBP = systolic blood pressure; SD = standard deviation. Ultrasound-assessed NAFLD cutoff = determined by the presence of a diffuse increase in fine echoes in the liver parenchyma compared to the kidney or spleen parenchyma.

**Table 6:** Sodium intake, age, BMI, and percentage of women in groups with the lowest and highest sodium intake.

Author-Separation of Groups	Sodium Intake (mg/d)		Age (Years) Mean ± SD		BMI (kg/m²)		% Female	
	Lowest	Highest	Lowest	Highest	Lowest	Highest	Lowest	Highest
Zhou et al. (2021) [19]–Quartile	2511	4258	52 ± 18	52 ± 18	28 ± 6	29 ± 7 *	54	49 *
van den Berg et al. (2019) [24]–Quartile	1889 ± 414	5061 ± 981	55 ± 13	52 ± 11 *	26 ± 4	28 ± 5 *	70	28
Choi et al. (2016) [21]–Quintile (Female)	1077	3310	38 ± 7	40 ± 8	21 ± 3	22± 3	-	-
Choi et al. (2016) [21]-Quintile, Male	1219	3485	39 ± 8	39.3 ± 7.9	24 ± 3	24 ± 3	-	-
Huh et al. (2015) [20]–Tercile	2416 ± 368	4324 ± 529	49 ± 16	55 ± 15 *	23 ± 3	25 ± 3 *	58	56 *
Emamat et al. (2021) [10]-Tercile	3183 ± 994	5143 ± 2966	44 ± 13.9	44 ± 14	29 ± 6	31 ± 8 *	59	57
Takahashi et al. (2022) [22]-Median	2960 ± 560	4520 ± 640	69 ± 10	65 ± 11 *	23 ± 4	25 ± 4 *	53	52
Luo et al. (2022)	?	?	?	?	?	?	?	?

Data as mean ± standard deviation or median. * *p* < 0.05. Values in mEq/d and mmol/d were transformed into mg/d by multiplying the value by 23. Choi et al. (2016) did not perform a statistical analysis of these parameters. Salt consumption was transformed into sodium consumption by dividing it by 2.5.

**Table 7:** Risk of bias in human studies.

Authors (Year)	Zhou et al. (2021) [19]	van den Berg et al. (2019) [24]	Choi et al. (2016) [21]	Huh et al. (2015) [20]	Emamat et al. (2021) [10]	Takahashi et al. (2022) [22]	Luo et al. (2022) [12]
Were the criteria for inclusion in the sample clearly defined?	Yes	Yes	No	Yes	No	No	Yes
Were the study subjects and setting described in detail?	Yes	Not clear	Not clear	Yes	No	Not clear	No
Was the exposure measured in a valid and reliable way?	No	Yes	No	No	No	No	No
Were objective, standard criteria used for measurement of the condition?	Yes	Yes	Yes	No	Yes	Yes	Yes
Were confounding factors identified?	Yes	No	Yes	Yes	No	No	No
Were strategies to deal with confounding factors stated?	Yes	No	Yes	Yes	No	No	No
Were outcomes measured in a valid and reliable way?	No	No	Yes	No	Yes	No	Yes
Was appropriate statistical analysis used?	Not clear	Not clear	Not clear	Not clear	Not clear	Not clear	Not clear

## 4. Discussion

In the present systematic review, we sought to understand the influence of sodium intake on NAFLD markers in humans and animals. Since the adverse effects of both high and low-sodium intake on metabolic health are described, the possibility exists that both negatively influence NAFLD development.

The human studies evaluated here systematically point to a positive association between sodium intake and NAFLD markers, particularly related to increased caloric intake, body weight gain, and insulin resistance development. Salt intake has been associated with an imbalance in the action of hormones that control appetite. Zhang et al. [25] found an important relationship between 24-h sodium excretion and fasting plasma ghrelin concentrations. Meanwhile, Lanaspa et al. [14] verified a reduced sensitivity to leptin in mice fed a high-salt diet. These effects together lead to hyperphagia, caloric intake, reduction in energy expenditure and, consequently, weight gain, all risk factors for the development of insulin resistance.

Insulin resistance is the cornerstone of NAFLD development, and the decline in insulin sensitivity in peripheral tissues, particularly accompanied by elevated compensatory hyperinsulinemia, promotes the uptake of free fatty acids by the liver and lipogenesis, leading to steatosis. Nonetheless, the effect of high- sodium intake on insulin sensitivity is still controversial, with studies showing negative [26], positive [27], or no effect [28]. Moreover, the positive association between sodium intake and NAFLD is weakened by the fact that most of the studies included were observational, a design not suitable for assessing causality.

It is expected that sodium intake covariates with energy intake, ultra-processed foods, sugary drinks, saturated fat, BMI, metabolic diseases, male gender, and socioeconomic status [14,29,30,31,32], among others. Therefore, it is difficult to know if the results obtained in studies with humans are a direct physiological effect of higher sodium consumption, or secondary to other associated factors. This can be observed in three studies [10,19,21], in which correction for BMI reduced, or even nullified, the effects of sodium intake. In this sense, incomplete control of confounding variables, as observed in most human studies (Table 5), impairs the interpretation of causality.

Concerns related to the quantification of sodium intake have grown in recent years, especially due to studies demonstrating increased all-cause and cardiovascular disease mortality with sodium restriction [33,34,35,36]. The International Consortium for Quality Research on Dietary Sodium/Salt [TRUE] does not recommend the use of questionnaires or spot and short duration (<24 h) timed urine for assessing individual sodium intake [37,38]. These methods have a low correlation with “real” sodium intake and could potentially distort the evaluated relationships. Furthermore, a single quantification of 24-h urinary sodium excretion does not adequately reflect sodium intake, requiring at least 3 non-consecutive measurements given the wide variation in sodium intake [37]. None of the studies in this review evaluated sodium intake through multiple assessments of 24-h urinary sodium excretion. Similarly, the use of predictive formulas for the diagnosis of NAFLD can bias the analysis since their components are associated with sodium intake.

During the preparation of this review, Shojaei-Zarghani et al. [5] published a systematic review with a meta-analysis evaluating the effect of sodium on NAFLD prevalence that mostly agreed with our thoughts. The main results of the study by Shojaei-Zarghani et al. [5] were: higher risk of developing NAFLD in high- sodium intake compared to low intake (OR = 1.6, 95% CI: 1.19–2.15); surveys using predictive formulas (FLI) to assess NAFLD showed a higher effect size (OR = 2.02, 95% CI: 1.29–3.17) than those assessing disease by FibroScan or ultrasonography (OR = 1.81, 95% CI: 1.24–2.65); studies with sodium excretion (24 h and <24 h) showed a higher risk of developing NAFLD (OR = 2.48, 95% CI: 1.52–4.06, I2 = 96.00%) compared with dietary assessments of sodium (OR = 1.23, 95% CI: 1.15–1.32). It is important to point out that these data refer to the uncorrected risk ratio and that the assessment of the certainty of the evidence (assessed by the GRADE method) was considered very low, so these data should be evaluated with great caution.

In addition, Lanaspa et al. [14] observed in a retrospective cohort the ability of sodium consumption to predict the development of NAFLD in individuals with energy consumption less than 2150 Kcal/day (the human study by Lanaspa et al. was not considered in this review due to a simple comparison between low and high-sodium consumption).

Regarding animal studies, the results suggest an association between sodium consumption and markers of NAFLD in the shape of U or J. Most of them, except for Cabrera et al. [7], agree that a higher sodium intake is harmful for NAFLD. Steatosis or TG concentration in the liver, and expression of inflammatory and fibrosis markers seem to be increased in animals fed a high-sodium diet. However, there also seems to be a negative effect of sodium restriction on NAFLD markers compared to animals on a diet with normal sodium concentration.

The high consumption of sodium leads to the development of NAFLD by increasing the endogenous production of fructose and oxidative stress, directing toward mitochondrial damage and leptin resistance [13,14]. This results in increased energy consumption, body weight gain, and supply of fatty acids to the liver, while impairing fat metabolism in the liver. SIRT3 is a crucial deacetylase that restricts the formation of reactive oxygen species in mitochondria. The *SIRT3* knockout in mice led to the development of fatty liver disease and metabolic syndrome due to the excessive acetylation of multiple mitochondrial proteins [39]. Gao et al. [17] showed that the reduction of *SIRT3* expression is an important mechanism for the development of NAFLD in mice fed a high-sodium diet.

Guo et al. [17] observed in mice that the offspring of mothers fed a high-sodium diet during pregnancy and lactation were more susceptible to the development of hepatic steatosis during the lactation period. The authors associated these results with changes in the gut microbiome.

**Figure 2:**
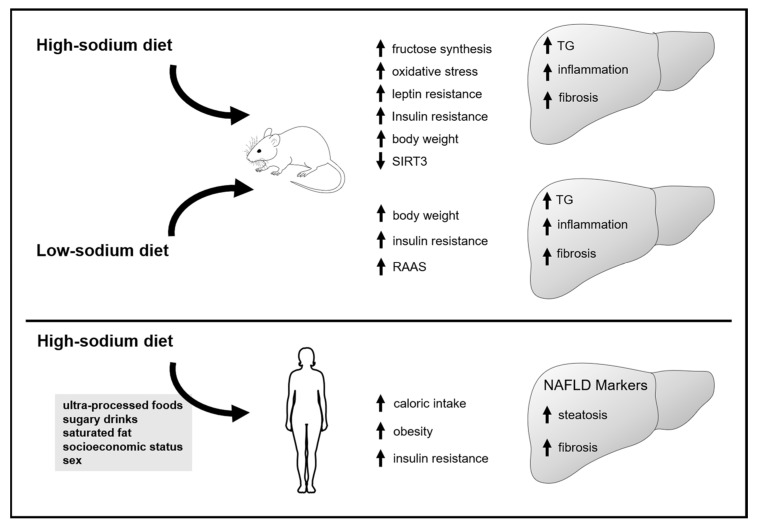
Major effects of salt consumption on NAFLD in animals and humans. In animal models, the direct effect of high-sodium intake has been robustly demonstrated, favoring the development of NAFLD. In most animal studies, markers of NAFLD have been found with evidence of increased lipogenesis, inflammation, and fibrosis. In high-sodium fed animals, these events are mediated by increasing the synthesis of fructose, oxidative stress, insulin and leptin resistances, and body weight, together with a reduction in sirtuin 3 (SIRT3). In a low-sodium diet, liver damage relates to the activation of the renin-angiotensin-aldosterone system (RAAS) and the induction of an insulin resistant state. On the other hand, high-sodium intake in the human population is positively associated with NAFLD markers, although it is also invariably associated with a higher caloric supply, together with industrialized and ultra-processed food intake, containing high sugar and high-fat amounts. There is also a contribution to sex and socioeconomic status. Altogether, this impacts body weight gain, and susceptibility to a chronic inflammatory status related to insulin resistance. From this point of view, variations in sodium intake may lead to liver damage by inducing lipogenesis and oxidative and inflammatory stress due to insulin signaling impairment in the liver and other organs. The Figure was partly generated using Servier Medical Art, provided by Servier, licensed under a Creative Commons Attribution 3.0 unported license.

On the other hand, the explanation of how sodium restriction contributes to NAFLD has been attributed to metabolic changes, such as increased body weight, plasma lipids, insulin resistance, and the RAAS. In the study by Cabrera et al. [7], the authors proposed that high-sodium intake leads to decreased expression of mineralocorticoid receptor and aldosterone, which allows overexpression of SIK1. SIK1 reduces the expression of lipogenic enzymes, diminishing steatosis. Conversely, animals on a low-sodium diet increases aldosterone levels and activate the mineralocorticoid pathway, leading to phosphorylation/inactivation of SIK1 and upregulation of lipid synthesis [7]. A summary of the effects of salt consumption on NAFLD in both animals and humans is depicted in Figure 2.

It is interesting to note that among interventions that evaluated a reduction in salt consumption, Kim et al. [15] observed an increase in the expression of inflammatory cytokines in the group with normal consumption compared to the group with moderately low sodium intake (0.4% Na^+^ vs. 0.13% Na^+^). It appears that in that case, a moderate restriction may have been protective, which was not seen with more intense restrictions.

The use of experimental animals has been a powerful tool in the understanding of several pathologies. Although they may not faithfully represent human physiology, animal studies are, in general, better controlled and allow us access to organs/tissues that are difficult to collect in humans, providing deeper insights into interventions. Many of the mechanisms that explain the relationship between low-sodium intake and the development of NAFLD in animals have already been observed in humans, such as the increased activity of the RAAS, insulin resistance, and disturbance in lipid metabolism [4]. However, why, so far, have studies in humans not shown a higher prevalence of NAFLD in lower sodium intake, similar to what was observed in murine models? It is possible to consider that (1) the pathophysiology of metabolic disorders associated with sodium intake in murine and human models is not identical; (2) the sodium concentration used in the feed of animals in the low-sodium groups is so low that it does not properly represent the current human sodium consumption; (3) uncontrolled confounding factors distort the relationship between sodium intake and NAFLD markers, requiring intervention studies to better understand the pathophysiology of sodium intake in humans. These questions must be answered by future studies.

This review has some limitations: Only one researcher carried out the research and screening of articles and data extraction, which increases the chance of errors; the research was carried out in only one database, so it is possible that articles not indexed in PubMed were not analyzed. It is possible that articles that evaluated NAFLD markers did not present the term NAFLD (or one of the similar terms introduced in the research) in their title or abstract, especially the articles on animals; this occurred with two of the seven articles on animals evaluated.

## 5. Conclusions

Liver steatosis, inflammation, and fibrosis are overwhelmingly demonstrated in experimental animal models on low- or high-sodium intake. On the other hand, in observational studies with humans, sodium consumption is positively associated with the development of NAFLD, although it is difficult to determine its independence from environmental factors, including the consumption of ultra-processed industrialized food, sugar-sweetened beverages, and high-fat diets.

## Figures and Tables

**Table 1:** Tool for critical analysis of cross-sectional studies–JBI and criteria for the judgment of each item.

1. Were the criteria for inclusion in the sample clearly defined?	
Yes	Clearly defined inclusion and exclusion criteria
No	Inclusion and exclusion criteria not clearly defined
2. Were the study subjects and the setting described in detail?	
Yes	Total population and groups described in detail, including: sociodemographic data, location, period of time, mode of selection or recruitment.
No	Description of the total population or groups lacking a lot of information.
Not clear	Description of the total population or groups lacking little information.
3. Was the exposure measured in a valid and reliable way?	
Yes	24-h urinary sodium excretion
No	24-h food recall; Food frequency questionnaire; < 24-h urinary sodium excretion
4. Were objective, standard criteria used for measurement of the condition?	
Yes	NAFLD diagnosis defined by diagnostic criteria existing in the literature.
No	others
Not clear	
5. Were confounding factors identified?	
Yes	Identified confounding factors: age, sex, energy consumption, dietary data, sociodemographic characteristics, alcohol consumption, smoking, physical activity, and metabolic diseases.
No	At least one unidentified confounding factor
6. Were strategies to deal with confounding factors stated?	
Yes	Confounding factors were used as exclusion criteria or included in the multiple logistic regression analysis. If it was not included, the author justified the non-inclusion.
No	At least one factor not included in multiple regression analysis
7. Were the outcomes measured in a valid and reliable way?	
Yes	ultrasound; FibroScan; nuclear magnetic resonance
No	Formula-based diagnostic
8. Was appropriate statistical analysis used?	
Yes	Analysis based on multiple regressions. The author details the method of choosing the covariates added to the model.
No	Simple comparisons between groups, simple correlations.
Not Clear	Analysis based on multiple regressions. The author did not detail the method for choosing the model covariates. The author added variables separately to the complete model.

Judgments were established by the author based on the JBI definitions for each item and the review objectives.

## Data Availability

No new data were created or analyzed in this study. Data sharing is not applicable to this article.
